# Widefield fluorescence lifetime imaging of protoporphyrin IX for fluorescence‐guided neurosurgery: An ex vivo feasibility study

**DOI:** 10.1002/jbio.201800378

**Published:** 2019-02-20

**Authors:** Mikael T. Erkkilä, Bianca Bauer, Nancy Hecker‐Denschlag, Maria J. Madera Medina, Rainer A. Leitgeb, Angelika Unterhuber, Johanna Gesperger, Thomas Roetzer, Christoph Hauger, Wolfgang Drexler, Georg Widhalm, Marco Andreana

**Affiliations:** ^1^ Center for Medical Physics and Biomedical Engineering Medical University of Vienna Vienna Austria; ^2^ Advanced Development Microsurgery, Carl Zeiss Meditec AG Oberkochen Germany; ^3^ Christian Doppler Laboratory for Innovative Optical Imaging and Its Translation to Medicine Medical University of Vienna Vienna Austria; ^4^ Institute of Neurology General Hospital and Medical University of Vienna Vienna Austria; ^5^ Department of Neurosurgery General Hospital and Medical University of Vienna Vienna Austria

**Keywords:** fluorescence guided surgery, fluorescence lifetime imaging, neurooncology, photodynamic diagnosis, time of flight camera

## Abstract

Achieving a maximal safe extent of resection during brain tumor surgery is the goal for improved patient prognosis. Fluorescence‐guided neurosurgery using 5‐aminolevulinic acid (5‐ALA) induced protoporphyrin IX has thereby become a valuable tool enabling a high frequency of complete resections and a prolonged progression‐free survival in glioblastoma patients. We present a widefield fluorescence lifetime imaging device with 250 mm working distance, working under similar conditions such as surgical microscopes based on a time‐of‐flight dual tap CMOS camera. In contrast to intensity‐based fluorescence imaging, our method is invariant to light scattering and absorption while being sensitive to the molecular composition of the tissue. We evaluate the feasibility of lifetime imaging of protoporphyrin IX using our system to analyze brain tumor phantoms and fresh 5‐ALA‐labeled human tissue samples. The results demonstrate the potential of our lifetime sensing device to go beyond the limitation of current intensity‐based fluorescence‐guided neurosurgery.

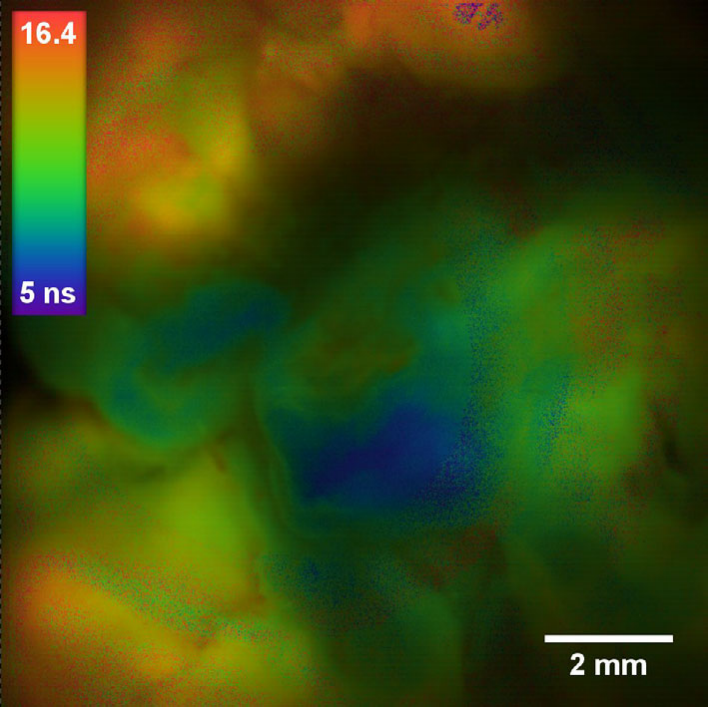

## Graphical abstract

To optimize the resection of gliomas during neurosurgery, we present an imaging system capable of wide field fluorescence lifetime mapping with 11 mm field of view and 250 mm working distance. Based on a time of flight dual‐tap CMOS camera and a modulated laser at 405 nm, we show unobserved quenching effects in tissue phantoms and demonstrate lifetime imaging on 5‐aminolevulinic acid (5‐ALA) labeled human ex vivo brain tumor samples.

## 1 INTRODUCTION

The discrimination between tumorous and healthy tissue in brain tumor surgery is of fundamental importance to achieve a maximal safe extent of resection and better patient outcome 1. Fluorescence‐guided surgery, based on 5‐aminolevulinic acid (5‐ALA) induced protoporphyrin IX (PpIX) 2, has shown to help the surgeon achieve more complete resections and prolonged progression‐free survival in malignant glioma patients 3. To detect even lower concentrations of PpIX which cannot yet be resolved by the surgeon using commercially available surgical microscopes, spectroscopic measurements 4, 5 or hyperspectral imaging 6 was proposed with promising results for the visualization of even low‐grade gliomas. In general, the measurement of fluorescence intensity is affected by the optical properties of the tissue which pose a major problem in PpIX quantification as these need to be characterized beforehand. As an alternative approach, we propose to monitor the fluorescence lifetime 7 instead of the intensity. The lifetime is an intrinsic property of the molecule; hence, its detection is not affected by light scattering or absorption. On the other hand, it is highly sensitive to tissue chemical micro‐environment and the fluorescence decay time of endogenous fluorophores such as nicotinamide adenine dinucleotides or flavins has been successfully used for label‐free tumor diagnostics 8. For PpIX, for example, the reported lifetimes range from 16 ns in solvent 9, 10 to 6 ns in prostate adenomacarcinoma cells 9 and to 3 ns in glioma cells 11 clearly indicating strong variations in different environments. The lifetime of non–5‐ALA‐labeled brain tumors in vivo, more specifically gliomas, has been reported using a point‐wise measuring probe by Butte et al. 12, a fiber‐bundle based endoscope with 4 mm field of view (FOV) by Sun et al. 13 as well as recently using a multiphoton micro‐endoscope with 350 microns scan range by Kantelhardt et al. 14. For an overview of lifetime imaging in neurosurgery the interested reader is referred to the review of Marcu and Hartl 15.

For real‐time neurosurgical guidance based on fluorescence lifetime, the imaging should be sufficiently fast to ultimately achieve real‐time visualization as well as allowing for a FOV and working distance of at least 10 and 200 mm, respectively. Finally, the technique should be optimized to image PpIX at its native lifetime (around 16 ns) and be mostly unaffected by ambient light in the surgical room. Based on these minimum requirements for surgical microscopes we screened the available technologies for lifetime imaging.

The gold standard of time‐correlated single photon counting (TCSPC) using photon counters, as recently demonstrated in vivo on human brain by Kantelhardt et al. 14, was omitted due to the slow imaging rate over a larger FOV and the risk of damaging the detectors by accidental illumination by ambient light. In addition, the high repetition rate of the ultrafast pulsed laser of 80 MHz is too fast to allow native PpIX to fully decay within the detection window Δ*t* = 1/(80 MHz) = 12.5 ns. Pulsed lasers could also be operated at lower repetition rates, for example, 20 MHz, but this would reduce the pixel dwell time equally by this factor. Direct temporal sampling as described by Eibl et al. 16 was also considered due to fast imaging speed. However, the photon budget is limited by the single pulse energy (100 nJ) which in return limits the detection low fluorophore concentration. Pulses in the *μ*J regime would be better suited but come at the price of lower repetition rate and more expensive light sources.

Wide field fluorescence lifetime imaging (FLIM) approaches using time gated intensified cameras were proposed by Sun et al. 13 and McGinty et al. 17 but were not widely used due to the high voltages and costs involved. Owing to rapid technological development of time‐of‐flight (TOF) sensors and cameras 18 as well as the requirements mentioned above, we developed a FLIM system for PpIX imaging in brain tumor tissue around a modulated dual tap complementary metal‐oxide semiconductor (CMOS) camera 19, 20. Specifications such as working distance, imaging rate and sensitivity were chosen to match the requirements for current neurosurgical microscopes. In this article, we show the feasibility of PpIX lifetime imaging using our TOF camera–based approach on brain tissue phantoms as well as on ex vivo human tumor samples and discuss the potential transfer of this technology into a surgical environment.

## 2 METHODS

### 2.1 Frequency domain lifetime imaging

In TOF sensors operating in the frequency‐domain, a laser is modulated with a duty cycle of 50% at a reference frequency *f*_*R*_ *,* and the beam is aimed at a target of interest. Light reflected from the target is then detected by a fast detector which is located next to the light source. To compute the distance *d* that the light has traveled from the laser over the target to the detector, the reference and detected signal are cross‐correlated and the phase delay ΔΦ with the best correlation is obtained. The distance *d* can then by calculated via *d* = *c*·ΔΦ/(2*πf*_*R*_) where *c* is the speed of light. In the case of a delayed response *τ* by the target (eg, fluorescence) the phase delay needs to be adjusted to:

(1) $$\Delta \Phi =2\pi f_{R}\left(\frac{d}{c}+\tau \right)$$

The first term corresponds thereby to the TOF contribution which needs to be known to obtain the fluorescence lifetime from a frequency‐based measurement. Although not as accurate as TCSPC, it allows the use of relatively inexpensive modulated diode lasers or light emitting diodes while operating similar to a lock‐in amplifier which discards any DC components such as ambient light. To obtain a lifetime map the laser beam is either scanned over the sample or a multi‐tap CMOS camera is used. In these cameras photoelectrons are stored in specific charge bins (taps) depending whether the excitation laser is on or off. Thereby, the ratio of electrons stored in the “laser on” ‐tap compared with the “laser off” ‐tap directly correlates to ΔΦ. In addition, the photon budget is now mainly limited by the exposure time. Hence, within the limits of negligible dark noise, longer exposure allows for adaptation to lower fluorescence intensity without the need for changing any components of the system.

### 2.2 Imaging system

**Figure 1:**
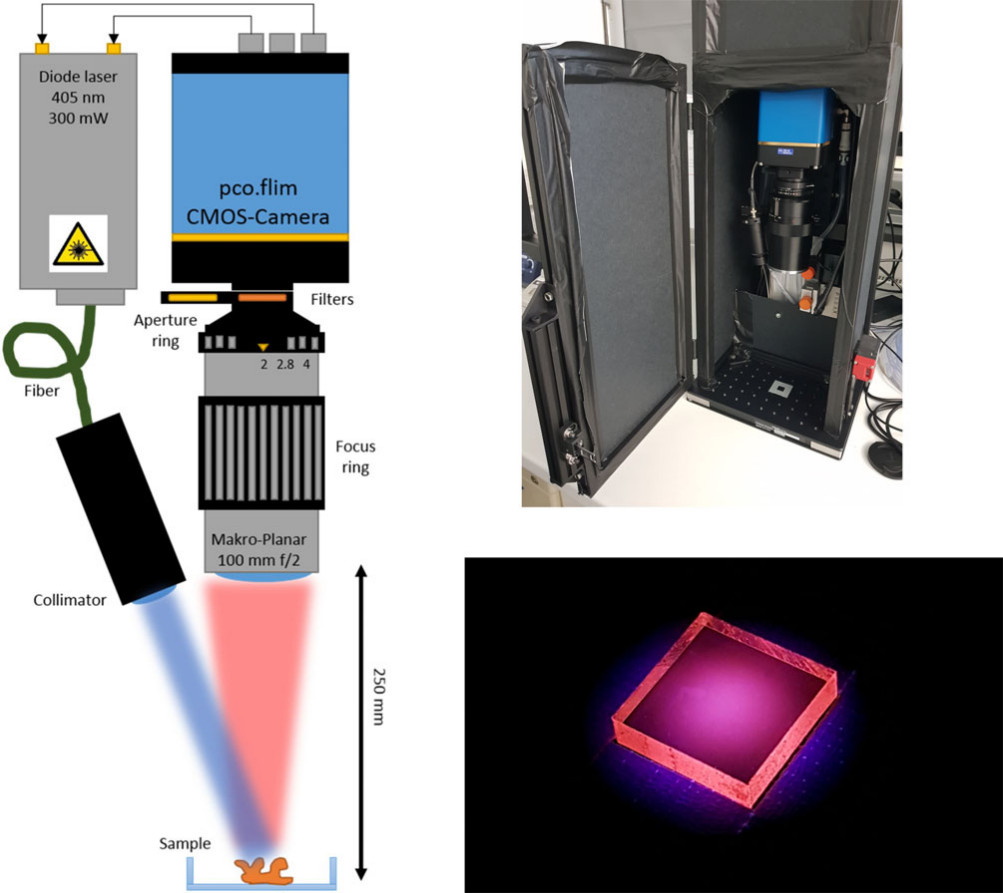
The amplitude‐modulated laser excites the sample, and the time‐delayed fluorescence is detected by the phase‐sensitive camera (left). Picture of enclosed system (right, top) and of the solid protoporphyrin IX (PpIX) reference sample (right, bottom)

A commercial dual tap CMOS camera (pco.FLIM, pco AG, GER) with 1008 by 1008 pixels and up to 90 frames per second was used as wide field TOF detector 19, 20. The sample was illuminated with 50 mW/cm^2^ irradiance by a collimated 405 nm diode laser (405‐300‐PhoxX+; Omicron Laserage GmbH, Rodgau, Germany) digitally modulated by the camera's internal signal generator. The modulation frequency was fixed to 10 MHz which is optimized for a lifetime of 15.9 ns. A macro photo lens (Makro‐Planar 100 mm/f2; Carl Zeiss AG, Oberkochen, Germany) and a fluorescence bandpass filter (FF‐665/150; IDEX LLC, Rochester, New York) were attached to the camera allowing a minimum FOV of 11.0 mm at 250 mm free working distance. The FOV could be adjusted by changing the working distance and refocusing with the photo lens. The object sided numerical aperture was determined to be 0.07 at a f‐stop of *f*/*#* = 2.0. The whole system (30 × 30 × 80 cm^3^) was completely enclosed and only accessible through an interlock‐secured door to ensure laser safe operation (see Figure 1). The exposure time was always kept *t*_exposure_ ≤ 200 ms to reduce dark noise and adjusted when necessary to avoid saturation of the detector during the acquisition. For the most accurate lifetime measurements, 16 intensity images were acquired equally spaced in phase delay over a full 2*π* range. Calibration with a reference target including exposure time bracketing 20 has to be performed prior to any measurements to eliminate intensity‐dependent phase artifacts and to obtain absolute lifetime values.

### 2.3 Reference target

To compensate for non‐fluorescent TOF contributions, the system is calibrated prior to any measurements with a solid state PpIX reference sample embedded in acrylic glass (12 × 12 mm^2^, custom made by Starna Scientific Ltd, Ilford, UK). As the lifetime might be altered due to the different chemical environment within the acrylic glass, the lifetime of the reference needs to be determined once. This value is then used repeatedly for the calibration procedure. The reference target is stored in the dark and is only illuminated during the procedure to avoid bleaching. To obtain the lifetime of the PpIX reference, the sample itself and a fluorescein solution were imaged with a TCSPC (Becker & Hickl, Berlin, Germany) laser scanning microscope (Carl Zeiss LSM 710). The same fluorescence bandpass filter (*λ*_*c*_ = 665 nm, Δ*λ* = 150 nm) was used to avoid the detection of shorter wavelength transitions. Fluorescein showed a single‐exponential decay with *τ*_*f*_ = (3.4 ± 0.2) ns while the PpIX sample exhibited a double‐exponential behavior with *τ*_1_ = (2.0 ± 0.3) ns and *τ*_2_ = (7.2 ± 0.4) ns for the fast and slow contributions, respectively. The fast lifetime contribution is due to the initiator used to induce the polymerization process which is by itself slightly fluorescing. To obtain a single reference lifetime for the PpIX sample, the TCSPC data was fitted with a mono‐exponential fit *τ*_0_ = (5.5 ± 0.2) ns. The result was validated with our FLIM system by referencing with the fluorescein solution at *τ*_*f*_ and measuring the lifetime of our reference to *τ*_*R*_ = (5.5 ± 0.8) ns.

### 2.4 Phantom design

Tissue phantoms composed of PpIX dimethly ester (CAS:5522‐66‐7; Sigma‐Aldrich, St. Louis, Missouri) dissolved in dimethyl sulfoxide (DMSO), lipid emulsion (Intralipid 20%), distilled water and yellow food coloring (McCormick) were generated based on the study of Jermyn et al. These phantoms are state of the art for the calibration of spectroscopic and hyperspectral PpIX imaging devices 6. The lipid emulsion simulated tissue scattering with *μ*_*s*_[cm^−1^] = {30, 35, 40, 45} while the yellow food color acted as absorber with *μ*_*a*_[cm^−1^] = {18, 30, 42, 50, 60} at 405 nm. Note that the food dye also generates background fluorescence which is specifically intended to simulate tissue autofluorescence. To imitate high and low‐grade glioma tissue three different concentrations (2500, 625, 156 ng/mL) of PpIX were mixed for every absorber and scattering value combination which resulted in overall 60 phantoms which were filled in cuvettes. To reduce photobleaching which results in reduced intensity and lifetime, the measurement time including sample placement and exposure adjustment was kept below 10 seconds for each sample.

### 2.5 Brain tissue samples

To demonstrate the feasibility of our approach for tumor imaging, we performed an ex vivo study with tissue samples routinely collected during 5‐ALA fluorescence‐guided surgeries. One patient with a recurrent astrocytoma and five glioblastoma patients undergoing surgery were enrolled. Informed consent was obtained according to national laws and regulations as well as approval by the ethics committee of the Medical University of Vienna (ethics approval number EK419/2008 ‐ Amendment 04/2018). To not interfere with the clinical workflow, the tissue resected was cut into two parts. The first part was sent directly to the neuropathology department for routine diagnostic workup while the second one was imaged with our system. The samples were stored in artificial cerebrospinal fluid and imaged within 1 hour after resection. During measurement, the tissue sample was placed on a quartz microscope slide and the laser was only emitting during image acquisition to minimize photobleaching. A lifetime map was obtained within 80 to 3200 ms depending on the set exposure time. In one patient with a deep seated glioblastoma, a tissue sample was collected from a surgical route to the tumor and this sample turned out to be normal tissue according to histopathological analysis.

## 3 RESULTS AND DISCUSSION

**Figure 2:**
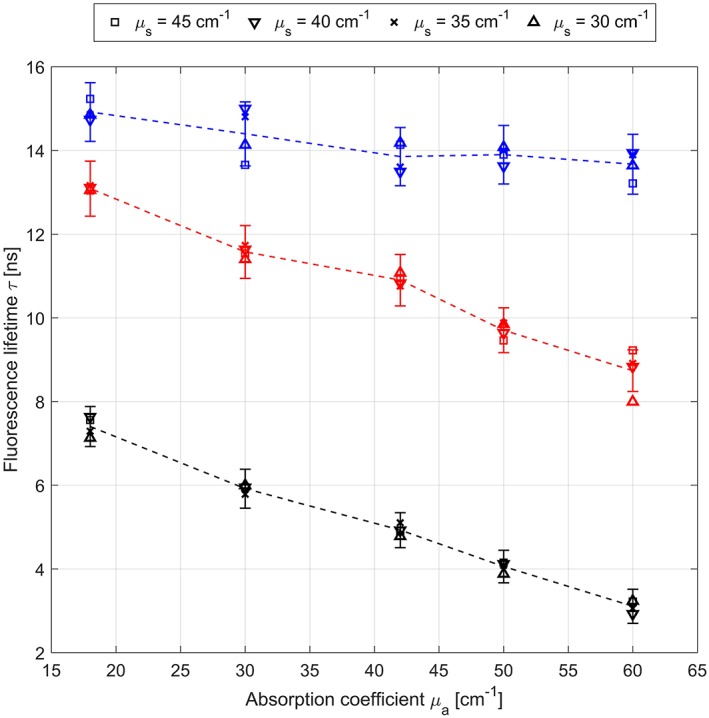
Fluorescence lifetime of tissue phantoms composed of protoporphyrin IX (PpIX) (dissolved in dimethyl sulfoxide [DMSO]), intralipid and yellow food color dye with 2500 ng/mL (blue), 625 ng/mL (red) and 156 ng/mL (black) PpIX concentrations. The symbols correspond to different scattering coefficients (equal to Intralipid concentrations) while the absorption coefficient is influenced by varying the concentration of the food dye

The fluorescence lifetime obtained from the phantom measurements for 2500, 625 and 156 ng/mL PpIX concentrations are presented in Figure 2. As the phantoms are homogeneous the average and SD were computed over the whole FOV. For all concentrations, a drop in lifetime is observed with increased absorption which is simulated by the yellow food dye. This behavior is enhanced toward lower PpIX concentrations. On the other hand, the influence of scattering simulated by intralipid is negligible within the error margins of the measurements. We first discarded the possibility of photon‐reabsorption of PpIX which would artificially increase the lifetime at higher PpIX concentrations as we did not expect an influence by absorption. Measurements with pure PpIX in DMSO with *τ* = (16.4 ± 0.6) ns and *τ* = (15.4 ± 0.8) ns at the highest (2500 ng/mL) and lowest concentration (156 ng/mL), respectively, show only small variations and are mostly within the error margins. This suggests that the food coloring induces additional interactions besides increased absorption. By evaluating the intensity changes relative to the food dye concentration, we observed both loss in intensity and reduced lifetime with increasing dye concentration. Although the lifetime should be unaffected by changes in tissue absorption, we conclude that the food dye quenches the PpIX fluorescence and thereby reduces the lifetime. In fact, the food coloring consists of two dyes, namely tartrazine and allura red, which are known to be strong quenchers 21, 22. We consider this an important observation as these phantoms are widely used for the calibration in spectroscopic imaging of PpIX in brain tumors 6 and quenching might generate false estimations of PpIX concentration values. To still use these phantoms to calibrate spectrometric PpIX imaging devices the influence of the quenching needs to be predicted. One solution would be to measure the change in PpIX fluorescence lifetime over a large range of quencher concentrations and compute the quenching constant according to the Stern‐Volmer equation. As this constant is equally suited to describe the variation in intensity, the spectroscopic measurements on phantoms could then be corrected based on the absolute concentration of food dye employed.

**Figure 3:**
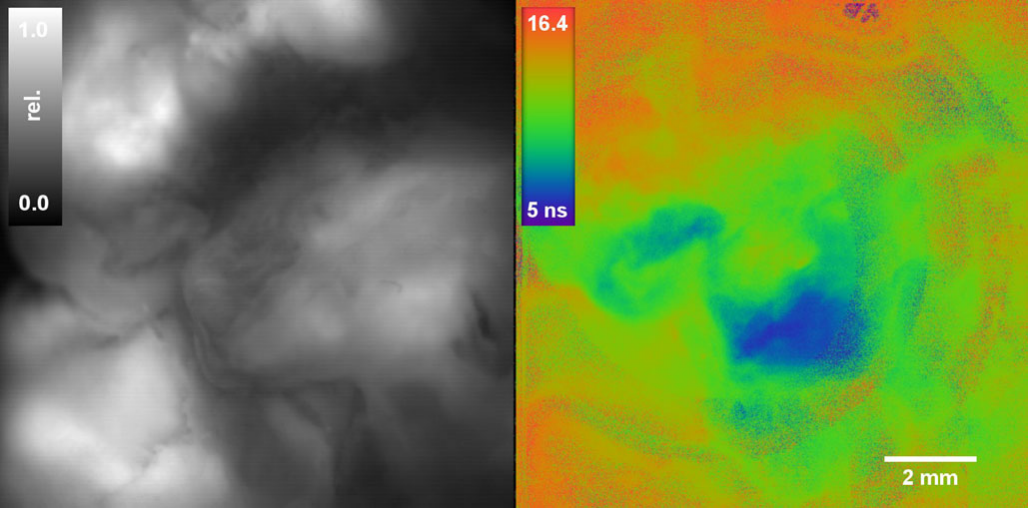
FLIM imaging of a 5‐aminolevulinic acid (5‐ALA) fluorescent tissue sample (1.1, *t*_exposure_ = 15 ms, see Table 1) from a glioblastoma with fluorescence intensity map (left) and lifetime map (right)

In Figure 3 we present, to our knowledge, the first ex vivo wide field lifetime imaging of fresh 5‐ALA induced PpIX labeled human glioblastoma (GBM) tissue. During surgery the neurosurgeon resected a strongly fluorescent tumor sample which was around 1 cm^3^ in size and showed typical anatomy with folds and fissures. The fluorescence intensity image shows strong fluorescence signal on the tissue surface where the incidence angle of the laser beam is nearly perpendicular. Tissue parts which are located in grooves or are strongly tilted relative to the illumination beam appear darker as the blue excitation light only penetrates the tissue superficially. As expected, the lifetime map is not affected by the intensity variations and reveals areas with long lifetime (around 16 ns) indicating native PpIX as well as shorter lifetime regions (from 12 to 5 ns) which could indicate the presence of quenched PpIX, photoproducts induced by photobleaching and other naturally occurring porphyrins (tissue autofluorescence). Comparing the lifetime with the histological findings of neuropathology (see Table 1), the short lifetime (blue, 5 ns) area most likely corresponds to necrotic tissue. As PpIX is produced within the heme cycle by the precursor 5‐ALA, necrotic tissue cannot contribute to the fluorescence. Thereby, the fluorescence seen is mostly due to non‐PpIX autofluorescence which exhibits a much shorter lifetime. This area is surrounded from longer lifetime areas (green, 10‐12 ns) which merge towards the edges into lifetime areas (red, 16 ns) of native PpIX indicating tumor cell infiltrations.

**Table 1:** Summary of lifetime *τ* and neuropathological results of the nine tissue samples obtained from six patients

Sample (diagnosis)^a^	Lifetime measurements			Neuropathology
	*τ* over FOV [ns]	*I*_max_ [norm.]	*t*_exposure_ [ms]	Description of histology
1.1–5‐ALA positive (Glioblastoma, grade IV)	12.19 ± 2.24	0.130	15	infiltration zone and compact tumor of moderate cellularity; high mitotic activity; small necrotic foci; vascular proliferations
2.1–5‐ALA negative (recurrent anaplastic astrocytoma, grade III)	3.12 ± 2.02	0.063	160	diffuse infiltration zone with transition into compact tumor of moderate cellularity; scar tissue; minor calcifications
2.2–5‐ALA pos./neg. (described in 2.1)	3.64 ± 2.30	0.047	160	moderately cellular tumor tissue and infiltration zone with tumor cell content; scar tissue
2.3–5‐ALA positive (described in 2.1)	10.14 ± 3.32	0.100	160	moderately cellular tumor tissue and infiltration zone with high tumor cell content; increased mitotic activity
3.1–5‐ALA positive (Glioblastoma, grade IV)	14.08 ± 2.19	0.066	20	tumor tissue of moderate cellularity; vascular thromboses
4.1–5‐ALA positive (Glioblastoma, grade IV)	15.10 ± 1.34	0.186	40	tumor tissue of moderate cellularity; small necroses; vascular thromboses
5.1–5‐ALA negative (Glioblastoma, grade IV)	5.32 ± 0.84	0.156	80	brain parenchyma with minimal tumor cell infiltration
5.2–5‐ALA positive (described in 5.1)	13.85 ± 1.48	0.088	5	brain parenchyma with some tumor cell infiltration
6.1–normal tissue	2.97 ± 2.43	0.082	200	normal tissue (minor micro‐calcifications)

Abbreviations: FOV, field of view; 5‐ALA, 5‐aminolevulinic acid.

The exposure time set on the camera is shown in column *t*_exposure_ while the maximum normalized intensity can be found in *I*_max_.

a 5‐ALA status as reported by the surgeons during fluorescence‐guided resection of the tissue sample. The histopathological diagnosis is established according to the current criteria of the World Health Organization (WHO).

**Figure 4:**
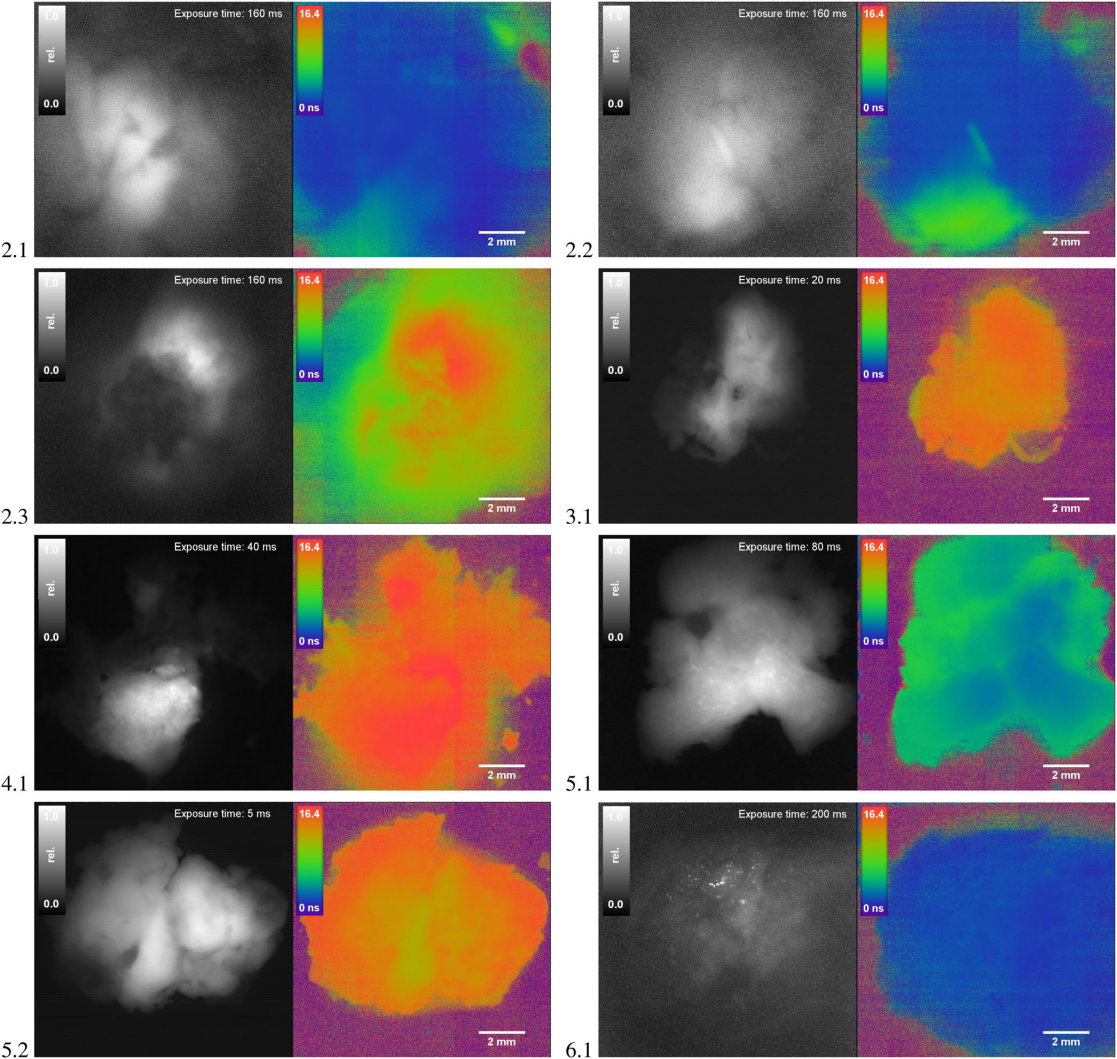
Fluorescence intensity (grayscale) and lifetime maps (color) of brain tumor samples (refer to Table 1). Within the lifetime maps areas outside of the sample are marked in purple color. Specimens 2.1 and 2.2 correspond to locally infiltrating tumor tissue (highlighted by increased lifetime, green) and are clearly distinguished from the shorter autofluorescence of the normal tissue. A focal glial cell infiltration can be seen in 2.3. Strong 5‐aminolevulinic acid (5‐ALA) positive fluorescence is seen in 3.1, 4.1 and 5.2 which is also clearly visible by the increased lifetime (red areas). In 5.1 a nonfluorescing part of a glioblastoma (GBM) shows minimal infiltrations of tumor cells. The intensity images are normalized for better contrast but were acquired with strongly varying exposure times

In Figure 4 we show eight tissue samples obtained from the five patients with the average lifetime, fluorescence intensity as well as camera exposure time and the report from neuropathology which is summarized in Table 1. Note that the lifetime was averaged over the whole tissue sample and might thereby lead to underestimated values as seen for samples 2.1 and 2.2. In general, strongly 5‐ALA fluorescent tissue samples like 1.1, 3.1, 4.1 and 5.2 show increased lifetimes above 12 ns and can be clearly distinguished from the normal tissue shown in 6.1 with a mean lifetime of around 3 ns. These samples correspond to glioblastoma patients and histological evaluation reveals the presence of high‐grade tumor tissue in all samples. In comparison to pure intensity imaging (shown in grayscale) the lifetime maps show the border of the sample much clearer. This is most evidently seen in tissue sample 4.1 where only a smaller part of the tissue seems to be fluorescent based on the intensity‐map while the lifetime map indicates that the whole sample is malignant tumor tissue.

In case of patient 5, sample 5.2 can be identified as high‐grade histology based on the increased lifetime and the 5‐ALA positive status by the surgeon which is supported by neuropathology. Sample 5.1 which was extracted during the same procedure was described by the surgeon as not fluorescent. By using a 16x exposure time, fluorescence could be detected, albeit it remained unclear whether this corresponded to PpIX‐ or auto‐fluorescence. However, the lifetime map shows values that were 2 ns longer than compared with the normal tissue 6.1 which indicated minor PpIX accumulations. The suspicion was substantiated by histological demonstration of diffuse tumor cell infiltrates in the tissue. We believe that this increased discrimination will help to improve resection of areas with diffuse tumor infiltration.

With patient 2, we demonstrate the ability of PpIX lifetime imaging on a recurrent astrocytoma which was first considered low‐grade glioma (grade II) before surgery. During the resection, the surgeon investigated the area under blue light illumination and detected a focal accumulation of slightly fluorescent tissue. This area was resected and is presented in sample 2.3 which was later found to be a small high‐grade focus of the underlying astrocytoma surrounded by a diffuse infiltration zone. Well into the surgical procedure, the ambient light was further decreased to allow better adaptation of the surgeon's eye to the low fluorescence intensity, and the tissue sample 2.2 with very low fluorescence visible as well as the adjacent non‐fluorescent sample 2.1 were resected. Imaging with our system revealed that the fluorescence intensity of samples 2.1 and 2.2 are nearly identical. In fact, when comparing the intensity and lifetime map, the contributions of short and longer lifetimes are almost nearly equal in intensity. We thereby suspect that the autofluorescence is as strong in these areas as the remaining PpIX fluorescence. The lifetime map shows additional contrast and can clearly point out areas of higher lifetime which in return most likely correlates to the histopathological report describing focal diffuse tumor cell infiltration. In sample 2.3 a slightly increased intensity was measured in a small isolated area with concurrent lifetime increase above 12 ns which is usually found in glioblastomas. Further studies need to be performed to evaluate if the lifetime of PpIX could potentially be used for intraoperative estimation of the tumor grade in glioma surgery.

The lifetime map in 1.1 also shows ring‐shaped artifacts, which are believed to be due to the laser excitation and the referencing procedure. As the laser exits through a single‐mode fiber, the gaussian beam shape leads to an uneven illumination. In addition, the PpIX reference target is 3‐mm thick and acts as parallel plate generating varying TOF contributions based on the incidence angle of the light. During referencing, the algorithm sets a reference phase delay for every pixel assuming a homogeneous surface with a single reference lifetime. When imaging a tissue sample this referencing procedure creates artifacts appearing in the lifetime map as locally occuring noise. Interestingly, this didn't occur for the other samples which were, however, smaller and flatter. Occasionally we also see stripes in the lifetime maps (see biopsy 2.1, 2.2 and 4.1) with slightly increased lifetime which occur randomly but do not affect the overall discrimination of normal and tumor tissue. These could be removed by taking the median of sequential measurements at the same position.

As to the question why PpIX lifetime differs between dense tumor tissue, moderate infiltrations and normal tissue, we hypothesize that PpIX is quenched by molecular oxygen within the cells. In general, PpIX fluorescence is known to be reduced when in contact with oxygen molecules 23. Furthermore, the partial oxygen pressure *pO*_2_ is decreased in brain tumor (ie, 10 mm Hg) compared with normal brain tissue (38 mm Hg) 24. This hypoxic property of tumor is well‐known and could be one possible explanation, besides the intrinsic difference in concentration, for the increased PpIX fluorescence visible in glioblastoma compared with low‐grade gliomas.

Finally, it is important to note that the ability to visually detect the fluorescence by the surgeon is highly subjective as can be seen in the differences between sample 2.3 (5‐ALA positive, 160 ms exposure time) and 5.1 (5‐ALA negative, 80 ms exposure time) which is one of the main motivation for finding are more objective mean of detecting PpIX fluorescence.

## 4 CONCLUSION

The proposed widefield fluorescence lifetime imaging system for 5‐ALA‐induced PpIX‐labeled brain tumors using a dual‐tap CMOS camera matches the requirements of neurosurgical microscopes with long working distances and large FOVs. In our phantom study, we observed strong quenching effects which need to be taken into account for intensity‐based PpIX concentration estimations. We propose to correct this effect by predicting the quenching using the Stern‐Volmer equation. The decrease in frame rate could be compensated by reducing the number of images acquired to compute the phase delay. Within our ex vivo study, we measured nine samples from six patients with tissue specimens from one astrocytoma and several glioblastomas as well as one normal tissue. In human gliomas, the lifetime is highly heterogeneous between compact tumor tissue, infiltration zones and normal brain parenchyma. Further investigations concerning the lifetime of PpIX in‐vivo and the relevance of photobleaching during surgery are required. It is important to note that malignant tissue exhibits an increased PpIX lifetime. Hence, the tumor border can be detected more accurately compared to intensity‐based imaging and might lead to an improved extent of resection and thus patient prognosis in glioma patients.

## CONFLICTS OF INTEREST

The authors declare no potential conflict of interests.
